# An Improved Magnetic Field Method to Locate the Grounding Conductor

**DOI:** 10.3390/s23083879

**Published:** 2023-04-11

**Authors:** Fan Yang, Songlin Liu, Yijun Lai, Jiayuan Hu, Shaohua Wang

**Affiliations:** 1 State Key Laboratory of Power Transmission Equipment and System Security and New Technology, Chongqing University, Chongqing 400044, China; 2 State Grid Zhejiang Electric Power Research Institute, Hangzhou 310014, China

**Keywords:** magnetic field differential method, optimal step size, rounding error, truncation error

## Abstract

The location of the grounding grid conductors is critical for performing corrosion diagnosis and maintenance work. An improved magnetic field differential method to locate the unknown grounding grid based on truncation errors and the round-off errors analysis is presented in this paper. It was proven that a different order of the magnetic field derivative can be used to determine the position of the grounding conductor according to the peak value of the derivative. Due to the accumulative error of higher differentiation, the truncation error and rounding error were used to analyze to accumulative error and to determine the optimal step size to measure and calculate the higher differentiation. The possible range and probability distribution of the two kinds of errors at each order are described, and the index of peak position error was derived, which can be used to locate the grounding conductor in the power substation.

## 1. Introduction

The grounding grid is an important piece of equipment that provides a common reference ground for various electrical equipment in the substation, quickly discharges the fault current in the event of a ground fault in the system, improves the ground potential distribution in the substation field, and ensures the safety of primary and secondary equipment and personnel under fault conditions [1,2]. The material of the grounding grid of the power system is mainly copper, which does not easily suffer from soil corrosion. In recent years, in Europe and the United States, steel gradually began to replace copper as the grounding grid material, but the steel grounding grid in operation for a relatively short period of time has not suffered serious corrosion. However, in China, India and other countries, since the conductors of the grounding grid are mostly made of steel, with the increase of the operation period, corrosion is prone to occur due to improper welding construction and the influence of geological conditions, and the conductors may become thinner or even broken [3,4,5], resulting in the grounding performance becoming reduced and the safety difficult to guarantee [6,7].

The grounding grid is buried in soil about 0.8 m deep underground, making it difficult to excavate and replace [8]. Therefore, predicting corrosion defects of the grounding grid based on information that can be obtained above the ground is important for guiding operation and maintenance [9,10,11] and has important engineering significance. In grounding grid research, generally, the optimization design of the grounding grid and the grounding performance of the grounding grid are studied [12,13]. In addition, many scholars have carried out research on grounding grid fault diagnosis, mainly based on electrical network theory [14,15,16] and electromagnetic field theory [17,18,19,20,21,22,23,24,25,26,27,28,29]. The method based on electrical network theory equivalently treats the branches of the grounding grid as pure resistances, injects current into the grounding grid, measures the conductor branch or node voltage of the grounding grid, studies and establishes a fault prediction diagnosis model, and uses optimization algorithms for solving [30,31,32,33]. These methods are based on the known topological structure of the grounding grid to perform grounding grid fault diagnosis. However, when diagnosing the grounding grid of a substation with a long history, sometimes the grounding grid drawing is missing or the actual structure of the grounding grid has a large error from the existing drawing due to reconstruction. In order to obtain the structure of the grounding grid, the grounding grid topology detection methods based on electromagnetic induction principle are widely used, mainly including the electrical source detection method (magnetic field method) [18,19,20,21,22,23,24,25,26] and the magnetic source detection method [27,28,29].

The magnetic source detection method uses a transmitting coil placed on the ground to pass a certain frequency of current. The induced currents generated by underground metal bodies under the excitation of the primary magnetic field produce changing magnetic fields in the surrounding space, called secondary magnetic fields. The position and orientation of underground metal conductors can be obtained by measuring the secondary magnetic field information received by the surface receiving coils. Magnetic source detection methods are divided into the frequency domain electromagnetic method and the time domain electromagnetic method according to different excitation and response characteristics. The time domain electromagnetic method is also called the transient electromagnetic method [27]. However, detection methods based on magnetic sources are greatly affected by metal structures.

The magnetic field method injects a sinusoidal current of a specific frequency into the grounding grid through the two upper guide wires of the grounding grid, measures the magnetic induction intensity generated by the current-carrying conductor of the grounding grid on the earth’s surface, and analyzes the distribution characteristics and laws of the magnetic induction intensity to determine the structure and fault states of the grounding grid [25]. Knowing the distribution of the magnetic field generated by the conductor of the current-carrying grounding grid on the surface, the inverse problem equation of the magnetic field can be established to solve the topological structure of the grounding grid. However, the inverse problem is usually ill-conditioned, it is difficult to obtain a unique solution or a stable solution, and complex regularization is required. The analysis method based on the magnetic field differential can avoid the solution of the inverse problem [34,35,36,37], but the error introduced in the numerical differential calculation may make the position of the grounding grid conductor deviate, and the selection of an appropriate measurement step is of great importance for the accurate determination of the conductor position.

In order to improve the reliability of the magnetic field differential method, this paper analyzed the range and probability distribution of the truncation error and rounding error produced by the numerical differential of the magnetic field method, derived the expressions of the mathematical expectation and variance of the differential main peak position error, and finally used Monte Carlo simulation to calculate the local optimal measurement step size under the second and fourth order differentials so that the magnetic field differential method reduced the total error and improved the accuracy of conductor positioning.

## 2. Error Analysis of Magnetic Field Differential Method

### 2.1. Magnetic Field Differentiation Method

The grounding grid is composed of regularly connected horizontal grounding conductors. The branch position information can be obtained by analyzing the distribution of the magnetic field generated by the current-carrying conductor branch. The rough outline of the grounding grid topology can be obtained by measuring the magnetic flux density distribution on the ground surface. However, due to the wide influence range of the magnetic field and the influence of the superposition of the magnetic field, it is not possible to accurately locate the conductor using the original magnetic field distribution characteristics. The magnetic field differential method can enhance the peak characteristics of the magnetic field through high-order differential and can improve the positioning accuracy of the grounding grid conductor. The magnetic field differentiation method is described below using a single current-carrying conductor model.

**Figure 1:**
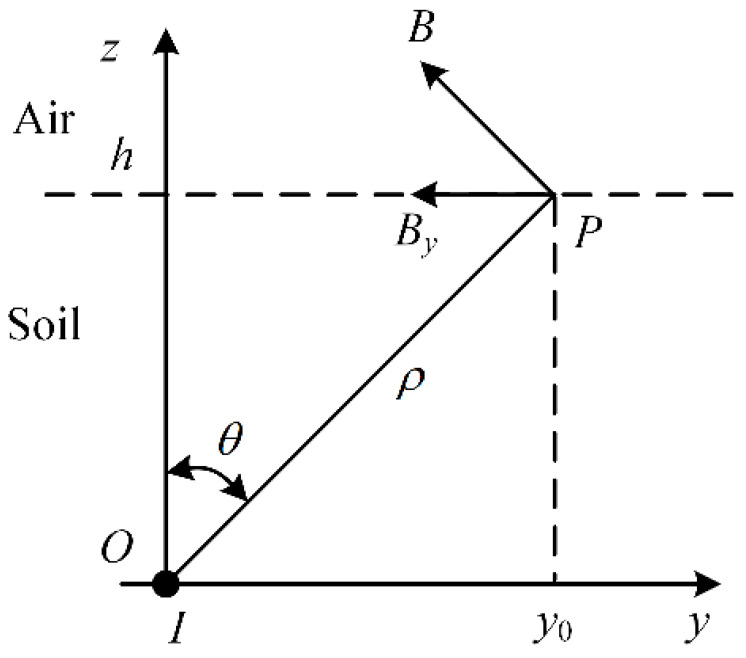
Single conductor current-carrying model.

The infinitely long conductor is placed on the *x*-axis through the coordinate origin, as shown in Figure 1; the conductor is buried horizontally in a single layer of uniform soil with a magnetic permeability *μ*; the buried depth is *h*; the current flowing through the conductor is *I*; and the direction of the current is vertical outward in the *y*-*z* plane. For point P on the ground surface, the vertical distance from the current-carrying conductor is *ρ*, and the angle between the line segment OP and the *z*-axis is *θ*.

According to the principle of potential continuity, the potential on both sides of the interface between the conductor and the soil is equal, and the resistivity of the conductor is significantly smaller than that of the soil. Therefore, the current density in the soil is significantly smaller than that inside the conductor, and the influence of the soil leakage current is negligible. Neglecting the leakage current of the conductor in the soil, the magnetic flux density generated by the current-carrying conductor at point P can be expressed by Ampere’s loop theorem as follows:

(1) $$B=\frac{\mu I}{2\pi \rho }e_{\phi }.$$

where $e_{\phi }$ is the unit vector in the direction perpendicular to the unit vector $e_{\rho }$, and $e_{\rho }$ is the unit vector in the direction of the line OP.

From the geometric relationship, it can be concluded that the magnetic flux density *B_y_*(*y*) parallel to the ground generated by the current-carrying conductor at point P is:

(2) $$B_{y}(y)=-\frac{\mu Ih}{2\pi }\frac{1}{h^{2}+y_{0}{}^{2}}.$$

Equation (2) describes the distribution of magnetic flux density in the horizontal direction generated by a single current-carrying conductor, which is called the shape function. For a grid-shaped grounding grid, the magnetic flux density distribution in the horizontal direction on the ground surface of the grounding grid can be equivalent to the superposition of the shape functions of each current-carrying branch of the grounding grid.

The higher the differential order of the shape function, the more complex its expression. Considering that the even-order derivative has the main peak characteristic, this paper calculated only the second-order differential and fourth-order differential of the shape function.

(3) $$B_{y}^{\left(2\right)}(y)=\frac{\mu Ih}{\pi }\frac{h^{2}-3y^{2}}{{\left(h^{2}+y^{2}\right)}^{3}},$$

(4) $$B_{y}^{\left(4\right)}(y)=-\frac{12\mu Ih}{\pi }\frac{h^{4}-10h^{2}y^{2}+5y^{4}}{{\left(h^{2}+y^{2}\right)}^{5}}.$$

**Figure 2:**
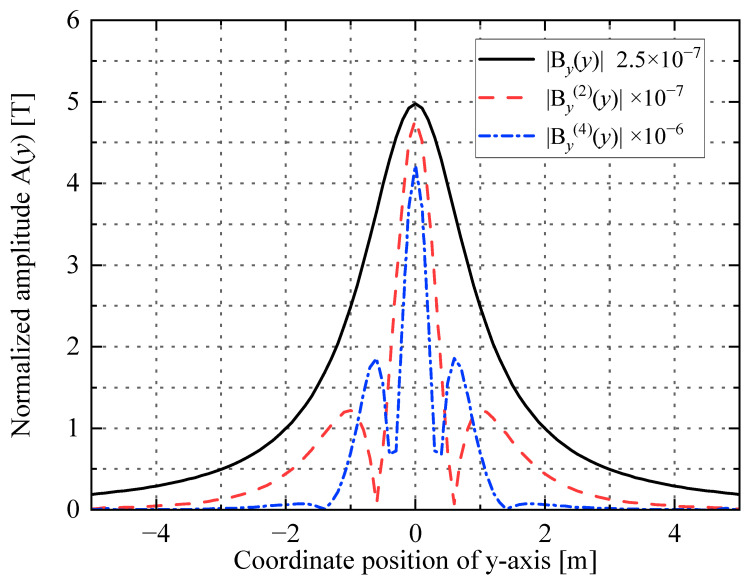
Curves of three shape functions.

When *I* = 1 A, *h* = 1 m, the curves of the shape function *B_y_*(*y*), the second-order differential $B_{y}^{\left(2\right)}$(*y*), and the fourth-order differential $B_{y}^{\left(4\right)}$(*y*) of the shape function are shown in Figure 2.

The main peak width in Table 1 is the width between the two zero points (or 1% of the main peak value) of the main peak. The side peak width is the width between the two zero points (or 1% of the main peak value) of the side peak adjacent to the main peak. The Widess resolution *R*_w_ is the ratio of the energy of the main peak maximum $b_{M}^{2}$ of the function to the total energy of the function *E*:

(5) $$R_{w}=\frac{b_{M}^{2}}{E},$$

where *b*_M_ is the maximum of the shape function

(6) $$E=\int_{-\infty }^{\infty }b^{2}(y)dy.$$

where *b* is the shape function.

From the comparison of the data in Table 1, it can be seen that, with the second-order and fourth-order derivatives of *B_y_*(*y*), the width of the main peak and the width of the side peaks gradually decrease, the total number of peaks and the Widess resolution gradually increase, and the signal recognition ability enhances. According to Equations (4) and (5), the positions of the main peaks of functions $B_{y}^{\left(2\right)}$(*y*) and $B_{y}^{\left(4\right)}$(*y*) are the same as those of the current-carrying conductors and are both at *y* = 0. Therefore, the positions of the main peaks of the second-order derivatives or fourth-order derivatives of the magnetic flux density *B_y_*(*y*) can be used to determine the locations of the grounding grid branches in the measurement area and thus to map the grounding grid topology.

### 2.2. Simulation of Current-Carrying Grounding Gird

**Figure 3:**
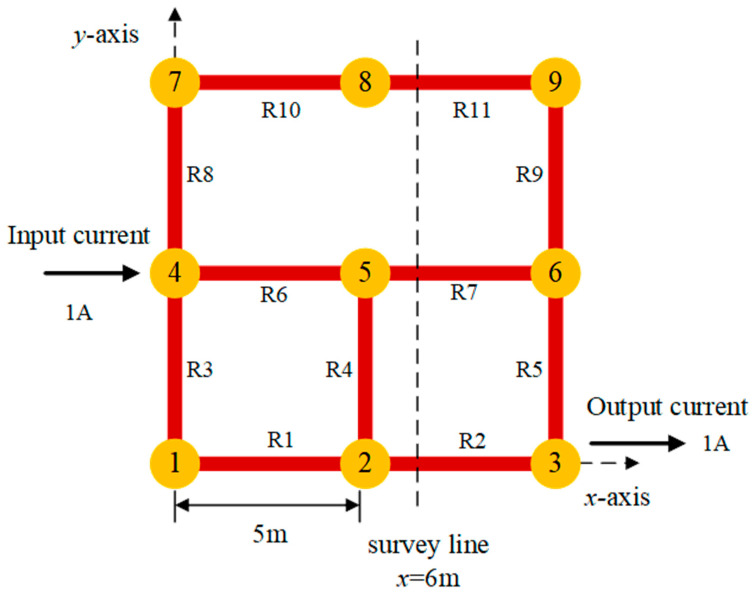
Simple current-carrying grid model.

As shown in Figure 3, a 2 × 2 grid of flat steel (cross-sectional area of 4 cm × 3 mm) was laid with a grid spacing of 5 m. The current of 1 A was injected from node 4 and flowed out from node 3. A Cartesian coordinate system *x-y-z* was established with node 1 as the origin of the coordinate axis, and the positive direction of the *z*-axis was perpendicular to the *x-y* plane upward. Below the plane *z* = *h*, there was a single layer of homogeneous soil with magnetic permeability *μ*. The magnetic permeability of the soil was approximated by taking the permeability *μ*_0_ in a vacuum. The resistivity of the conductor was significantly larger than that of the soil, and the soil leakage current had a negligible effect on the simulation results of the magnetic flux density. Therefore, we set a typical value of 80 Ω·m for the soil resistivity.

**Figure 4:**
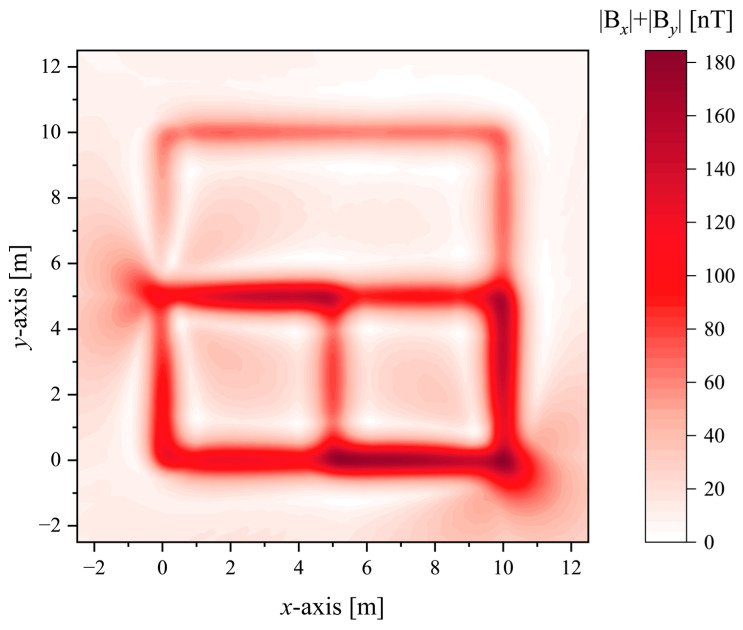
The distribution of the magnetic flux density mode at the plane *h* = 0.5 m.

**Figure 5:**
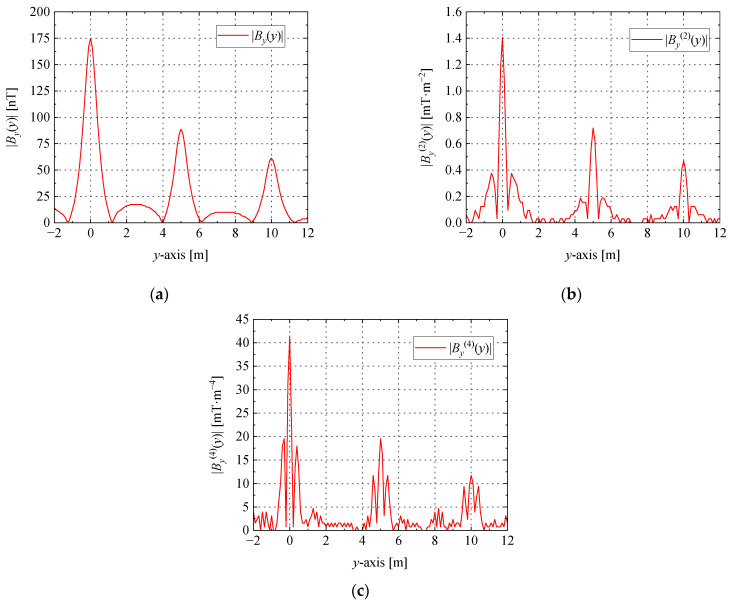
The curve of magnetic flux density differential at the survey line *x* = 6 m: (**a**) the absolute value curve of magnetic flux density; (**b**) the second-order differential absolute value curve of the magnetic flux density; (**c**) the fourth-order differential absolute value curve of the magnetic flux density.

Through the shape function of a single current-carrying conductor, it can be known that the peak values of the horizontal components *B_x_* and *B_y_* of the magnetic flux density can reflect the conductor position in different directions; thus, the modulus of the magnetic flux density is more important than its direction for the positioning of the conductors. |*B_x_*| + |*B_y_*| can reflect all current-carrying conductors in the *x*-*y* direction. In order to study the magnetic flux density generated by the current-carrying grounding grid, the simulation was performed using MATLAB based on the finite element method. The magnetic flux density |*B_x_*(*x*,*y*)| + |*B_y_*(*x*,*y*)| generated by the current-carrying grid branch was detected in the horizontal plane at a distance of *h* = 0.5 m from the *x-y* plane, while |*B_y_*(*y*)| was detected on the survey line at the position *x* = 6 m, and the results are shown in Figure 4 and Figure 5.

The results of the simulation are shown in Table 2. The grid spacing defined in the simulation is 5 m, and the errors of the grid spacing according to functions |*B_y_*(*y*)|, |$B_{y}^{\left(2\right)}$(*y*)|, and |$B_{y}^{\left(4\right)}$(*y*)| are 2.18%, 0.75%, and 0.28%, respectively.

### 2.3. Numerical Differential Error Analysis

When performing the position measurement of the grounding grid conductor, due to the limitations of the measurement equipment size, measurement time consumption, and other factors, it was not possible to measure a sufficient number of data points at the substation; thus, the horizontal component of the magnetic flux density measured on a certain survey line was a discrete sequence. When the differential method is used to locate the grounding conductor, a numerical calculation method is required. However, since the measured information did not contain the function expression and the noise introduced in the measurement process was unavoidable, the calculation result obtained by the difference quotient did not have high reliability, and the accuracy of the difference quotient result depends on the step size of the difference, i.e., the measurement interval. Sometimes, a small difference step size may lead to a large calculation error [38]. Therefore, it is necessary to analyze the sources of error in the process of numerical differentiation. In the following, the error situation of the magnetic field differential is analyzed from the perspective of truncation error and rounding error.

The formula for the central difference quotient commonly used In numerical differentiation can be expressed as:

(7) $$G(x)=\frac{f(x+d)-f(x-d)}{2d}.$$

where *d* is the differential step size.

Substituting *f*(*x*_0_ ± *d*) into formula (7) after performing Taylor expansion at *x* = *x*_0_, the truncation error is:

(8) $$\left|f^{\prime }(x_{0})-G(x_{0})\right|\leq d^{2}M/6,$$

where $M\geq \underset{\left|x-a\right|\leq d}{\max}\left|f^{\left(3\right)}(x)\right|$, and *a* is the center of the interval over which the maximum value of *f*^(3)^(*x*) is taken.

From the perspective of truncation error, the smaller the step size *d*, the more accurate the calculation result of numerical differentiation.

Considering the truncation error in the process of magnetic field differentiation, for the current-carrying grid shown in Figure 3, the survey line *x* = *x*_0_ is selected, the coordinates of the measurement starting point are (*x*_0_, *y*_0_), and the position of the *k*-th measurement point along the survey line on the *y*-axis is noted as *y_k_* = *y_0_* + *kd*, where *d* is the measurement interval. The nth order difference quotient of the y-direction component of the magnetic flux density *B_y_*(*y*) can be expressed as:

(9) $$B_{t}{}^{\left(n\right)}(y_{k})=\frac{B_{t}{}^{\left(n-1\right)}(y_{k+1})-B_{t}{}^{\left(n-1\right)}(y_{k-1})}{2d}\;,$$

where the order *n ≥* 1, when *n* = 1, *B_t_*(*y_k_*) = *B*(*y_k_*).

The truncation error due to numerical differentiation can be expressed as the difference between the difference quotient and the differential quotient:

(10) $$E_{t}^{\left(n\right)}(y_{k})=B_{t}{}^{\left(n\right)}(y_{k})-B^{\left(n\right)}(y_{k}).$$

When performing numerical difference calculations, the difference of two approximately equal numbers can result in a significant loss of valid numbers. The input point f(x+d) of the difference quotient is denoted as $\hat{f}(x+d)$, and the error between the input point $\hat{f}(x+d)$ and the real value f(x+d) is denoted as *ε*.

(11) $$G(x)=\frac{\hat{f}(x+d)-\hat{f}(x-d)}{2d}.$$

The error between the real value of the nth-order derivative *f*^(*n*)^(*x*) of function *f*(*x*) and its numerical calculation result can be expressed as:

(12) $$\delta (f^{\prime }(x))=f^{\prime }(x)-G(x)=\frac{\varepsilon_{2}-\varepsilon_{1}}{2d}+\frac{h^{2}}{6}f^{\left(3\right)}(a).$$

where a∈(x−d,x+d). If $\varepsilon_{m}=\max\{|\varepsilon_{1}|,|\varepsilon_{2}|\}$ is defined, the upper bound *E_r_*_max_ of the rounding error can be expressed as:

(13) $$E_{r\max}=\frac{\varepsilon_{m}}{d}.$$

Therefore, from the perspective of rounding error, a step size *d* that is too small will result in a large rounding error.

### 2.4. Peak Position Error Analysis

In order to accurately locate the grounding grid conductor, it is necessary to determine an optimal measurement interval *d*. The mathematical expectation *M*(*d*) and variance *D*(*d*) of the peak deviation can be used to describe the degree of the conductor positioning error, which is derived below.

First, consider the impact of the randomness of the measurement points on the position error of the main peak. The survey line *x* = *x*_0_ is selected, the coordinates of the measurement starting point are (*x*_0_, *y*_0_), and the position of the *k*-th measurement point along the survey line on the *y*-axis is noted as *y_k_* = *y*_0_ + *kd*, where *d* is the measurement interval. The probability density function of the truncation error *E_t_*(*y_k_*) at *y_k_* is *p_t_* = 1/*d*; thus, the randomness of *E*(*y_k_*) is determined by the rounding error.

Let the probability density of the rounding error *E_r_* at a measurement point be *p_r_*, and *p_r_* under the same measurement step *d* and order *n* is not affected by the location of the measurement point; thus, *p_r_* is a function of *E_r_*, where *E_r_* is a uniformly distributed random variable on [−*E_r_*_max_, *E_r_*_max_]. Then:

(14) $$p_{r}(E_{r})=\left\{ \begin{array}{l} \begin{array}{cc} \frac{1}{2E_{r}{}_{\max}}, & \left|E_{r}\right|<E_{r}{}_{\max} \end{array}\\ {\begin{array}{cc} 0, & \left|E_{r}\right|>E \end{array}}_{r}{}_{\max} \end{array}\right..$$

At the *n*-th order, *E_r_* is obtained by accumulating the two rounding errors *E_r_*_1_ and *E_r_*_2_ of the *n*-1 order, and then $p_{r}^{\left(n\right)}(E_{r}=\frac{E_{r_{1}}+E_{r_{2}}}{2d})$ can be expressed as:

(15) $$p_{r}^{\left(n\right)}(E_{r})=\int_{-\infty }^{\infty }2d\cdot p^{\left(n-1\right)}(E_{r_{1}})p^{\left(n-1\right)}(2dE_{r}-E_{r_{1}})dE_{r_{1}}.$$

**Figure 6:**
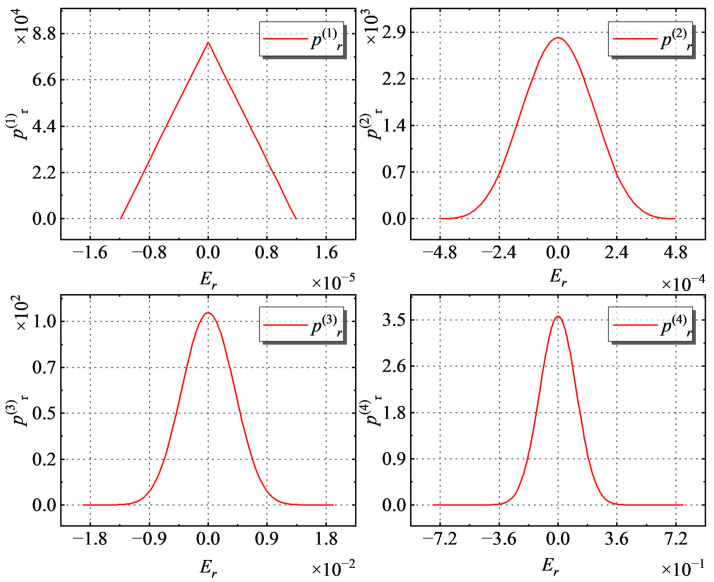
When *n* = 1~4, the distribution of $p_{r}^{\left(n\right)}$ on *E_r_*.

When *n* = 1~4, the distribution of *p*(*n*) *r* with respect to *E_r_* is shown in Figure 6.

The total error *E*(*y_k_*) is the sum of the truncation error and rounding error; thus, the probability density function *p*(*E*(*y_k_*)) at *y_k_* can be expressed as:

(16) $$p(E(y_{k}))=p_{t}p_{r}(E_{r}(y_{k}))=\frac{p_{r}(E(y_{k})-E_{t}(y_{k}))}{d}.$$

The error at *y_k_* is *E*(*y_k_*) and is denoted as event *k*; each event *k* is independent of each other, and the probability density function of events 1, 2,…, *m* being established at the same time is $\prod_{k=1}^{m}p(E(y_{k}))$, where the value range of *E*(*y_k_*) is as follows:

(17) $$E_{t}(y_{k})-E_{r\max}(y_{k})\leq E(y_{k})\leq E_{t}(y_{k})+E_{r\max}(y_{k}).$$

The peak position of the measured and calculated value *B_C_*(*y*) of the *n*-th order derivative of the magnetic flux density is denoted as *y_pC_*. Due to the presence of errors, there is a deviation between *y_pC_* and the peak position *y_pR_* of the real value *B_R_*(*y*). The deviation Δ*y_p_* is the difference between the calculated value and the real value, i.e., Δ*y_p_* = *y_pC_* − *y_pR_*. The probability density function $\prod_{k=1}^{m}p(E(y_{k}))$ is used to derive the mathematical expectation of the main peak position error.

First, consider the mathematical expectation of the offset caused by the error *E*_1_ at *y*_1_ when the errors of other points are constant. Assuming that *E*_1_ takes only s discrete values, *E*_10_, *E*_10_ + Δ*E*_1_, *E*_10_ + 2Δ*E*_1_,…, *E*_10_ + (*s* − 1)Δ*E*_1_, it corresponds to the distribution of a total of *s* function errors on *y*, and the cumulative expectation is:

(18) $$\sum_{j=1}^{s}p(E_{10}+(j-1)\Delta E_{1})\Delta y_{p1j}\Delta E_{1}.$$

Extending *E*_1_ from the discrete distribution to the case of continuous distribution, i.e., s→∞, the above formula becomes:

(19) $$\underset{s\to \infty }{\lim}\sum_{j=1}^{s}p(E_{10}+(j-1)\Delta E_{1})\Delta y_{p1j}\Delta E_{1}=\int_{E_{\max-}(y_{1})}^{E_{\max+}(y_{1})}p(E_{1}(y_{1}))\Delta y_{p1}dE_{1}$$

Equation (19) represents the mathematical expectation of the offset due to the error *E*_1_ at *y*_1_ when the error at other points is constant.

*Y*_q_ = *p*(*E*(*y*_1_))Δ*y_p_*_1_. *ξ*(*y*_1_) is the result of integrating $\prod_{q=1}^{m}p(E(y_{q}))\Delta y_{p}$ over *E*(*y_q_*), i.e.,

(20) $$\xi (y_{1})=\int_{E_{\max-}(y_{m})}^{E_{\max+}(y_{m})}\cdots \int_{E_{\max-}(y_{1})}^{E_{\max+}(y_{1})}Y_{q}dE(y_{1})\cdots dE(y_{m})$$

Equation (20) represents the expectation obtained by taking all possible cases of *E*(*y*_1_), *E*(*y*_2_), …, *E*(*y*_m_) at *y*_1_.

Notice that Δ*y*_p_ is affected by all *y_i_*; thus, the above equation cannot be expressed in terms of $\prod_{i=1}^{m}\int_{E_{\max-}(y_{1})}^{E_{\max+}(y_{1})}Y_{q}dE(y_{1})$.

Finally, consider that *y*_1_ can be varied within the first interval segment [*y*_min_, *y*_min_ + d). In summary, the mathematical expectation *M*(*d*) of the main peak deviation can be expressed as:

(21) $$M(d)=\int_{y_{\min}}^{y_{\min}+d}\xi (y_{1})dy_{1}$$

At the same time, the degree of dispersion of *Y_q_* should also be considered; thus, the variance *D*(*d*) of the main peak position error caused by d can be expressed as:

(22) $$D(d)=\int_{y_{\min}}^{y_{\min}+d}\int_{E_{\max-}(y_{m})}^{E_{\max+}(y_{m})}\cdots \int_{E_{\max-}(y_{1})}^{E_{\max+}(y_{1})}{\left(Y_{q}-M(d)\right)}^{2}dy_{1}\cdots dy_{m}dx_{1}$$

## 3. Experimental Analysis

### 3.1. Simulation Experiment

The time complexity of the algorithm used to calculate the main peak position error expectation *M*(*d*) and the main peak position error variance *D*(*d*) is exponential time complexity O(*k^n^*). It is difficult to obtain results quickly through computation, but the constructed and described stochastic process and probability distribution are completely accurate models; thus, the Monte Carlo simulation can be used to obtain approximate results of the problem. The method of the Monte Carlo simulation increases the number of trials, and if the test results converge when the number of trials is sufficient, the final converged value is used as the simulation result.

**Figure 7:**
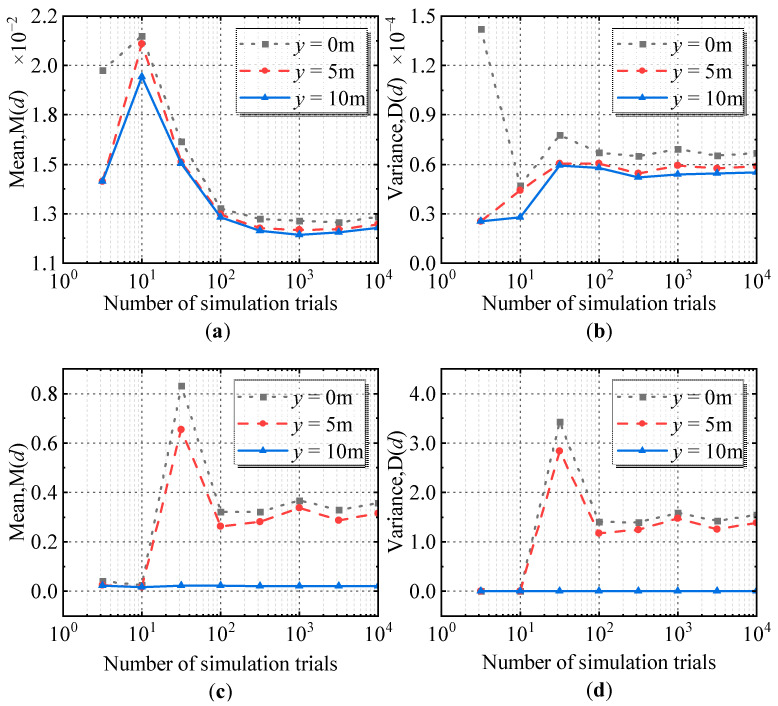
The expectation and variance of the main peak position error at the three conductors (*d* = 0.05 m): (**a**) Expectation *M*(*d*) of the main peak position error at the second-order differential; (**b**) Variance *D*(*d*) of the main peak position error at the second-order differential; (**c**) Expectation *M*(*d*) of the main peak position error at the fourth-order differential; (**d**) Variance *D*(*d*) of the main peak position error at the fourth-order differential.

First, the mean peak position error *M*(*d*) and the variance of the peak position error *D*(*d*) were calculated at a given measurement step *d*. Taking the current-carrying grid in Figure 3 as an example, the selected survey line was *x* = 6 m and the measurement step *d* was 0.05 m. The expectation *M*(*d*) and the variance *D*(*d*) of the main peak position error at the second-order differential and the fourth-order differential for the three conductors (*y* = 0 m, *y* = 5 m, *y* = 10 m) were calculated using Monte Carlo simulations, and the results are shown in Figure 7.

It can be seen from Figure 7 that the error expectation and variance of the main peak position at the three conductor positions (*y* = 0 m, *y* = 5 m, *y* = 10 m) gradually tended to converge as the number of simulation tests increased; thus, the convergence value can be used as the expectation and variance of the main peak position error of the simulation.

**Figure 8:**
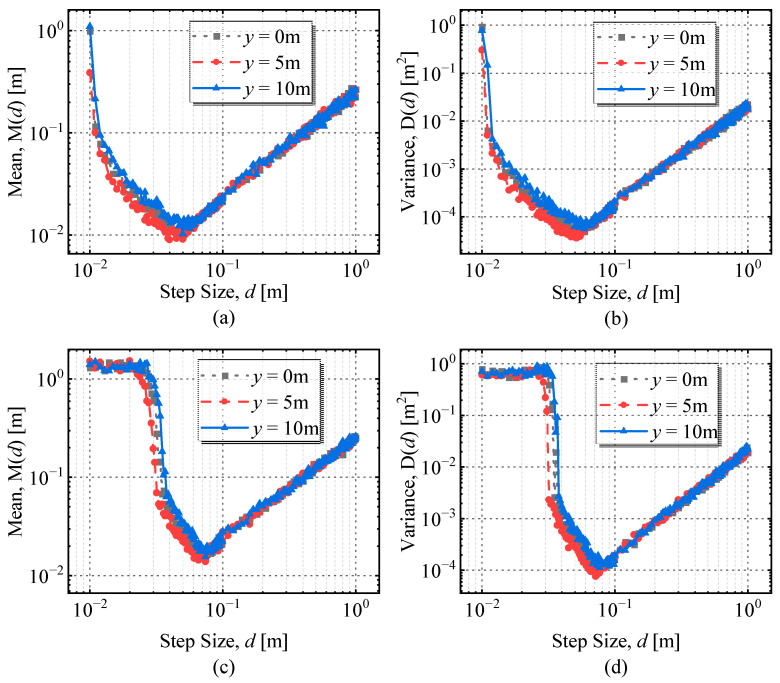
The expectation and variance of the main peak position error at the three conductors (*d* = 0.01~1 m): (**a**) Expectation *M*(*d*) of the main peak position error at the second-order differential; (**b**) Variance *D*(*d*) of the main peak position error at the second-order differential; (**c**) Expectation *M*(*d*) of the main peak position error at the fourth-order differential; (**d**) Variance *D*(*d*) of the main peak position error at the fourth-order differential.

Then, Monte Carlo simulations were used to calculate the expectation *M*(*d*) and variance *D*(*d*) of the main peak position error at three conductor locations (*y* = 0 m, *y* = 5 m, *y* = 10 m) with different measurement steps along the survey line *x* = 6 m, where the measurement step *d* takes values from 0.01 m to 1 m. When the number of simulation experiments was 10^4^ times, the expectation and variance of the main peak position error for the second-and fourth-order differentials of the magnetic field at each measurement step *d* are shown in Figure 8.

According to Figure 8, the second-order differentiation, the main peak position error expectation, and variance diverged when *d* was close to 0, and they reached the minimum when *d* = 0.04 m and gradually increased with the increase of *d*. At this time, the local optimum value of the measurement step was 0.04 m. Similarly, when the order was 4, the local optimal value of the measurement step was 0.06 m.

Finally, to confirm the generalizability of the locally optimal step size derived from the *x* = 6 m survey line, several survey lines were selected at different locations to calculate the main peak position error expectation *M*(*d*) and variance *D*(*d*) using the Monte Carlo simulation. The survey line *x*_0_ was taken in the range of [0 m, 10 m], and each time *x*_0_ was increased by 2 m. The calculation results are shown in Table 3.

If the magnetic field differentiation method is used to locate the grounding grid conductors at the power substation and it is desired to minimize the impact of the numerical differential calculation error on the conductor location, an optimal measurement step needs to be selected. According to the results in Table 3, the measurement step size *d* = 0.04 m or *d* = 0.05 m can be selected as the optimal measurement step size.

### 3.2. Substation Field Experiment

In order to verify the effectiveness of the optimal step size selection, a field experiment of substation grounding grid conductor positioning was carried out. In the experiment, three different measurement steps were used to measure the magnetic flux density on the surface along the survey line, and the differential method was used to calculate the second-order and fourth-order differentials of the magnetic flux density at each step.

**Figure 9:**
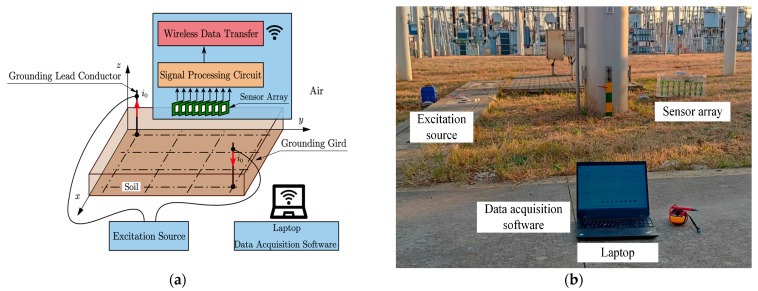
The 500 kV substation field experiments: (**a**) schematic diagram of conductor positioning experiment; (**b**) field experiment photo.

The schematic diagram of the conductor positioning experiment is shown in Figure 9a. The current output of the excitation source was connected to grounding lead conductors at the diagonal position in the test area. Eight PCB coils formed an array of magnetic field sensors with adjustable spacing. The signal conditioning circuit filtered and amplified the output signal of the magnetic field sensor. An 8-channel, 24-bit ADC (ADS1278) was used to convert the analog signal from the signal conditioning circuit into a digital signal. The ESP32 microcontroller communicated with the ADC through the SPI interface and sent the converted digital signal to the laptop via Wi-Fi. A photo of the experimental site is shown in Figure 9b.

**Figure 10:**
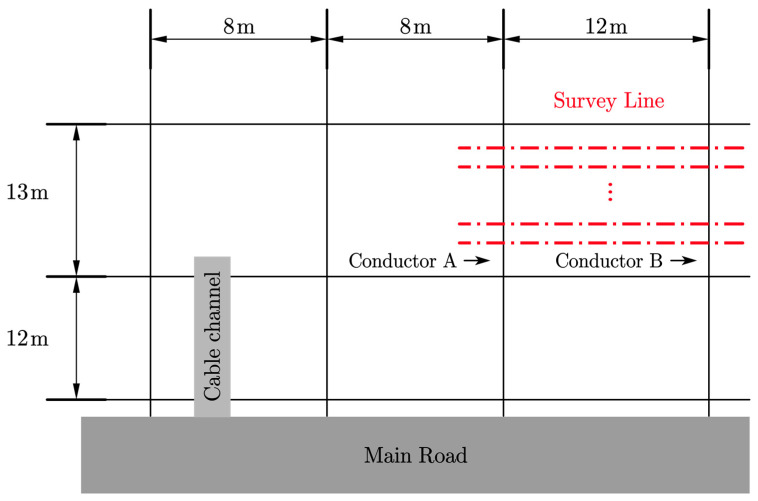
Schematic diagram of survey lines and topology.

Figure 10 shows a schematic diagram of survey lines and the topology of part of the substation’s grounding grid. The spacing of the ground grid conductors in the experimental area was unevenly distributed. The measurement area is shown in Figure 10, where the red dotted lines indicate the survey lines. Ten survey lines were laid out with a line spacing of 0.5 m. Each line was 20 m long, with a measurement point spacing of 0.02 m, 0.05 m, and 0.10 m. The sensing array was measured along the survey lines to record data at all locations. Two conductors, A and B, whose approximate positions were known, were located below the survey line.

**Figure 11:**
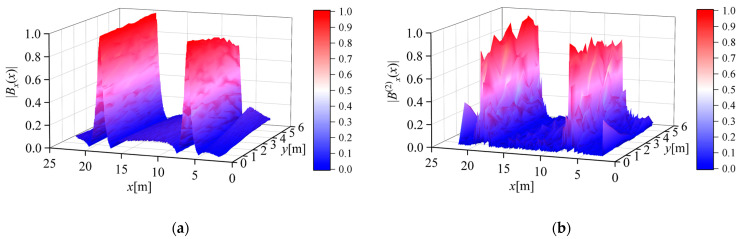

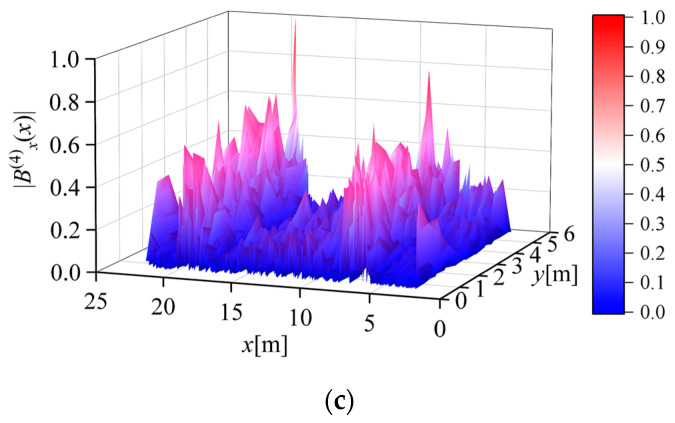
Normalized magnetic flux density absolute value measurement results when the measurement step size *d* = 0.02 m: (**a**) magnetic flux density; (**b**) second-order differential of the magnetic flux density; (**c**) fourth-order differential of the magnetic flux density.

Figure 11a shows the normalized absolute value of the magnetic flux density measured along the survey line when the measurement step length *d* = 0.02 m. Figure 11b,c shows the normalized absolute values of the second- and fourth-order differentials of the magnetic flux density calculated from the measurement results, respectively. It can be seen from the figure that the calculation result of the second-order differential can improve the Widess resolution and maintain the peak characteristic of the conductor position. However, due to the influence of numerical differential error and random noise in the measurement process, the calculated value of the fourth-order differential did not have good peak characteristics, and it is difficult to distinguish the position information of the conductor from the result.

**Figure 12:**
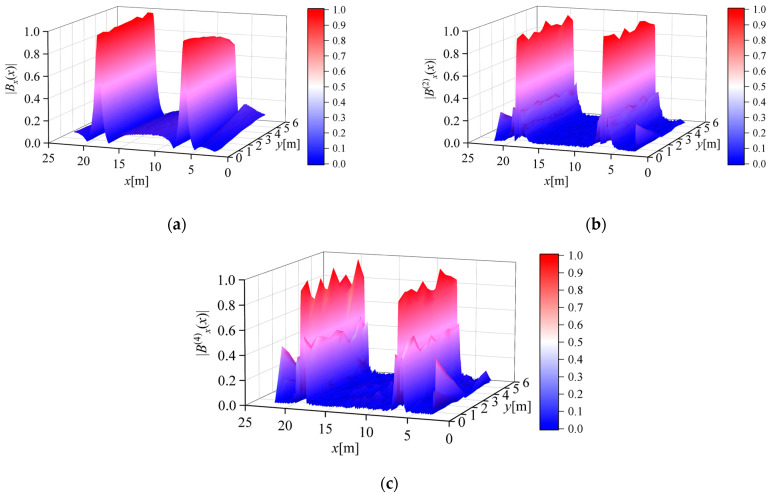
Normalized magnetic flux density absolute value measurement results when the measurement step size *d* = 0.05 m: (**a**) magnetic flux density; (**b**) second-order differential of the magnetic flux density; (**c**) fourth-order differential of the magnetic flux density.

Figure 12a shows the normalized absolute value of the magnetic flux density measured along the survey line when the measurement step length *d* = 0.05 m. Figure 12b,c shows the normalized absolute values of the second- and fourth-order differentials of the magnetic flux density calculated from the measurement results, respectively. It can be seen from the figure that the calculation results of the second-order differential and fourth-order differential can both improve the Widess resolution and maintain a good conductor position peak characteristic, and the calculation results reflect the actual position of the conductor.

Combined with the conclusion in Section 3.1, the local optimal measurement step *d* can be selected as 0.04 m or 0.05 m. Comparing Figure 11c and Figure 12c, it was found that, due to the influence of rounding errors, the fourth derivative of the magnetic flux density when the measurement step *d* = 0.02 m, compared with *d* = 0.05 m, the peak characteristic was completely lost, and the conductor was difficult to locate through the differential result. Comparing Figure 11b and Figure 12b, it was found that, due to the influence of rounding errors, the peak characteristic fluctuation of the second derivative of the magnetic flux density was more obvious when the measurement step length *d* = 0.02 m compared with *d* = 0.05 m, and the impact of measurement errors on the calculation results was more significant. This result is consistent with the conclusion stated in Section 3.1.

The measurement and calculation results when the measurement step length *d* = 0.10 m were close to the situation when *d* = 0.05 m, which can improve the Widess resolution while maintaining the peak characteristics. The measurement and calculation results under this condition are no longer listed separately.

In addition, the integer multiple decimation of the measurement data under the condition of *d* = 0.05 m can obtain part of the original measurement data under the condition of a larger measurement step. Numerical differential operation was performed on the extracted data, and its peak characteristics and Widess resolution were close to the case of *d* = 0.05 m. However, as the extraction multiple increased, the error between the measured value and the actual value of the conductor peak position gradually increased.

In summary, the experimental results are in good agreement with the simulation conclusions in Section 3.1; that is, there exists a local optimum value for the measurement step, and the selection of a suitable measurement step can reduce the error of conductor positioning.

## 4. Conclusions

An improved magnetic field method was proposed to locate unknown grounding grid conductors. The influence of the truncation error and rounding error of the magnetic field differential on the positioning of grounding grid conductors was studied, and the optimal measurement step size under different differential orders was given. By selecting the optimal measurement step size, the accuracy of the magnetic field differential method for locating the conductor of the grounding grid was improved. Through theoretical analysis and experimental verification, the following conclusions were drawn:

- A small measurement step causes a large rounding error in the numerical differentiation. With the increase of the measurement step, the truncation error caused by the numerical differentiation increases the error of the conductor positioning.
- The peak position deviation expectation and variance showed a trend of first decreasing and then increasing with the increase of the measurement step size. The measurement step size under different differential orders had a local optimum value, and the range of the local optimum value was given; this result is verified by simulation and experimental results.
- Choosing a reasonable measurement step size for different differential orders helps to improve the positioning accuracy of the grounding grid conductors.

The error analysis method in this paper expands the possibility of using the magnetic field differential method to reduce the error of grounding grid conductor positioning. At the same time, the results of this paper can provide a reference for the structural design of the magnetic field measurement sensor array, and the grounding grid conductor positioning instrument designed by the results of this study will have a higher conductor positioning accuracy, which is beneficial to the fault diagnosis work of large-scale substations.

## Figures and Tables

**Table 1:** Comparison of function shape properties.

Function	Main Peak Width (m)	Side Peak Width (m)	Widess Resolution
|*B_y_*(*y*)|	19.90	-	0.6361
|$B_{y}^{\left(2\right)}$(*y*)|	1.1552	3.3739	1.6849
|$B_{y}^{\left(4\right)}$(*y*)|	0.6504	1.0516	2.2847

**Table 2:** Simulation results of grid size.

Function	Calculated Grid Spacing (m)	Side Peak Width (m)
|*B_y_*(*y*)|	4.8912	2.18
|$B_{y}^{\left(2\right)}$(*y*)|	4.9623	0.75
|$B_{y}^{\left(4\right)}$(*y*)|	4.9861	0.28

**Table 3:** Local optimal step size at different survey line positions.

*x*_0_/m	0	2	4	6	8	10
Step size (2nd)	0.04	0.04	0.03	0.03	0.03	0.03
Step size (4th)	0.06	0.05	0.05	0.05	0.05	0.05

## Data Availability

Not applicable.
